# A simple device for delivering a capsule endoscope

**DOI:** 10.1055/a-2109-1343

**Published:** 2023-07-27

**Authors:** Wenbin Wu, Xianhong Zhao, Wanfeng Zheng, Beiping Zhang

**Affiliations:** Department of Gastroenterology, The Second Affiliated Hospital of Guangzhou University of Chinese Medicine, Guangzhou, Guangdong, China

Capsule endoscopy is a convenient and minimally invasive method for gastrointestinal visualization. Ingestion of the capsule however, is sometimes difficult for infants, children, and adults with dysphagia. The complication of capsule aspiration is increasingly reported, especially in patients with dysphagia 1 . The AdvanCE capsule endoscope delivery device has been approved for capsule endoscopy in such cases 2 . However, to our knowledge, the AdvanCE delivery device is not available to most medical institutions in China. Therefore, we developed a simple device for capsule endoscope delivery.

Herein, we report a case of a 22-year-old woman with the chief complaint of recurrent abdominal distension who was prepared for capsule endoscopy to examine the small bowel. However, she could not swallow the capsule, and we did not have access to the AdvanCE delivery device. Therefore, we developed a simple delivery device using available materials. The delivery device consisted of a conventional gastroscope, a transparent cap, and a transparent film glove. The transparent cap was fixed onto the end of the gastroscope. We then made a “pocket” for the capsule by cutting off a finger portion of the transparent film glove and putting the capsule inside it. A foreign-body forceps was then pushed through the biopsy channel to the end of the endoscope, and used to clamp the open end of the capsule pocket. Then, the forceps were gradually drawn back inside the biopsy channel, so that the capsule was half inside the transparent cap ( Video 1 ). Because of the loss of visualization, we used the transnasal gastroscope that approached the patient’s pharynx as it advanced, and we slowly advanced the delivery device under the direct vision of the transnasal gastroscope and pushed the capsule into the esophagus ( Fig. 1 ). The real-time display device of the capsule endoscope could also be used to assist in identifying the direction of the gastroscope. The capsule was pushed into the stomach, where the transparent film capsule “pocket” was dislodged using the foreign-body forceps. A snare was then used to push the capsule into the duodenum.

**Video 1** A novel method to deliver a capsule endoscope into the esophagus under the view of a transnasal gastroscope, in a patient unable to swallow the capsule.


**Fig. 1:**
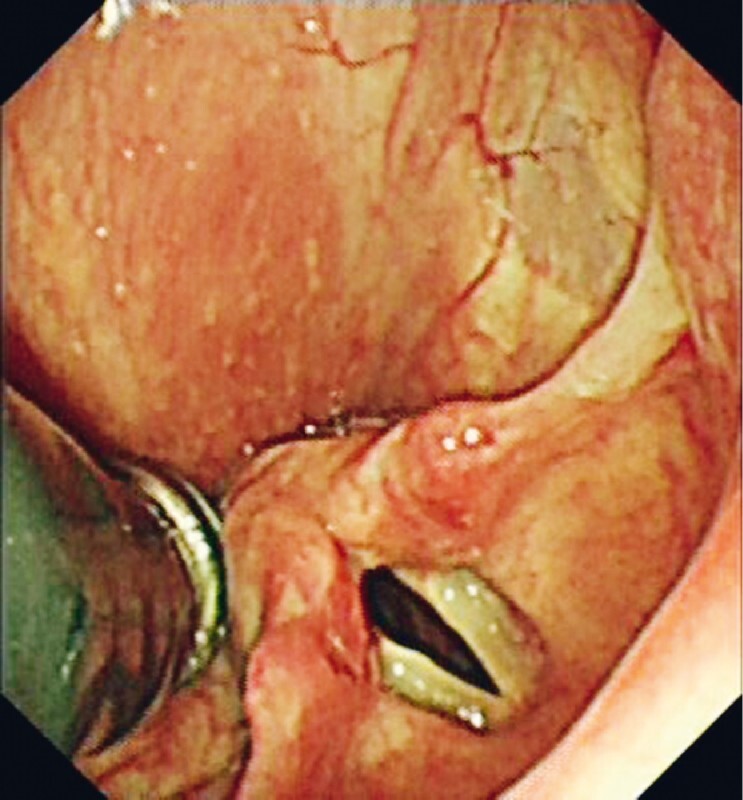
View as a transnasal gastroscope is used to deliver a capsule endoscope in a patient unable to ingest the capsule.

In conclusion, we have devised a method that can be safely and effectively used for capsule delivery in patients who cannot ingest the capsule endoscope.

Endoscopy_UCTN_Code_CPL_1AI_2AB

## Notes

Endoscopy E-Videos

https://eref.thieme.de/e-videos
*E-Videos* is an open access online section of the journal *Endoscopy* , reporting on interesting cases and new techniques in gastroenterological endoscopy. All papers include a high-quality video and are published with a Creative Commons CC-BY license. Endoscopy E-Videos qualify for HINARI discounts and waivers and eligibility is automatically checked during the submission process. We grant 100% waivers to articles whose corresponding authors are based in Group A countries and 50% waivers to those who are based in Group B countries as classified by Research4Life (see: https://www.research4life.org/access/eligibility/ ). This section has its own submission website at https://mc.manuscriptcentral.com/e-videos
